# Root and Root Canal Morphology Classification Systems

**DOI:** 10.1155/2021/6682189

**Published:** 2021-02-19

**Authors:** Mohmed Isaqali Karobari, Ayesha Parveen, Mubashir Baig Mirza, Saleem D. Makandar, Nik Rozainah Nik Abdul Ghani, Tahir Yusuf Noorani, Anand Marya

**Affiliations:** ^1^Conservative Dentistry Unit, School of Dental Sciences, Universiti Sains Malaysia, Health Campus, Kubang Kerian, Kota Bharu 16150, Kelantan, Malaysia; ^2^General Dental Practitioner, Hyderabad 500028, Telangana State, India; ^3^Conservative Dental Science Department, College of Dentistry, Prince Sattam Bin Abdulaziz University, Al Kharj, Saudi Arabia; ^4^Department of Orthodontics, University of Puthisastra, Phnom Penh, Cambodia

## Abstract

**Introduction:**

While there are many root morphology classification systems with their own distinct advantages, there are many shortcomings that come along with each system.

**Objectives:**

The aim of this review was to compare the various root and root canal morphology classifications, their advantages, limitations, and clinical and research implications. *Data Sources and Selection*. An extensive literature search was conducted on PubMed and Scopus to identify the published data on root and root canal classification systems published until 1 May 2020 using keywords, root canal classification system, classification systems for root canals, and root morphology. The related literature was reviewed and then summarized. *Data Synthesis*. Several studies have analysed and detailed root and root canal classifications and further added new subsystems, works of Weine et al. (1969) and Vertucci et al. (1974). Besides, Sert and Bayirli (2004) added supplementary types to Vertucci's classification system. A new classification was most recently introduced by Ahmed et al. (2017) involving the use of codes for tooth numbering, number of roots, and canal configuration.

**Conclusions:**

Weine et al. classified only single-rooted teeth, without considering multirooted teeth and complex configurations. Vertucci's classification included complex configurations, with Sert and Bayirli adding further complex supplemental types. Ahmed et al.'s classification simplifies classifying root and canal morphology while overcoming the limitations of several previous classification systems making it beneficial for implementation in dental schools.

## 1. Introduction

Every clinician aims to achieve the best treatment outcomes of endodontically treated cases. To attain the best results, one must diagnose the condition very well. Therefore, complete knowledge of root canal morphology with comprehensive understanding of the root canal system complexity is particularly important in clinical practice to reach the desired treatment goals. Additionally, sound knowledge of the root canal morphology classification is important for documentation and ease of communication amongst practitioners. While there are many root morphology classification systems, there are many shortcomings that come along with each system. While some are elaborate, there are others that are concise but do not provide enough information.

Apart from normal root canal morphology, a practitioner should have sound knowledge of possible root canal morphologic variations such as a single canal bifurcating into two canals that recombine to form a single canal later [1, 2]. This review was prepared with the aim to analyse various classification systems for root and root canal morphology and discuss their associated advantages, limitations, and research implications.

## 2. Methods

A general search was carried out to identify the published data on root and root canal morphology classification systems on PubMed and Scopus until the 1^st^ of July 2020 using keywords, root canal classification system, classification systems for root canals, and classification systems for root canal morphology. Several root and root canal morphology classification systems with modifications and supplemental types were identified, which are discussed in this review.

Several authors have given classifications on root canal configurations. Weine et al. [3] were the first to classify root canal morphology within a single root. They further added an additional type in the year 1982 [4]. In the year 1974, Vertucci et al. recognized further complex root canal systems and reported 8 types of configuration according to the pattern of division in the main root canal from leaving the pulp chamber to the apex of the root [5]. Sert and Bayirli in the year 2004 further added fourteen supplemental types to Vertucci's classification system [6]. A new classification was given by Ahmed et al. in the year 2017 [7], which is simple, easy to understand, and more accurate at classifying root canal configurations compared to earlier systems. The benefit of this new classification was that it was planned using codes that were easy to use by students and dentists alike. It was based on a coding system with individual codes for the tooth number, the number of roots, and the configuration of the canals. Subsequently, in the year 2020, Ahmed et al. carried out another study on the incorporation of the new root canal classification system and found out that the new system was more accurate and favoured by the final year undergraduate students in Malaysia [8].

A study was carried out by Ahmed and Dummer in 2018 to introduce a new classification system to classify tooth, root, and even accessory canal anomalies that could be used in conjunction with other systems for comprehensive analysis [9]. In the year 2020, Ahmed et al. carried out further research on the application of their previously published classification system in primary dentition and described the challenges of using this system [10]. Buchanan et al. studied the premolar anatomy extensively using two classification systems, and they concluded that the system given by Ahmed at al. delivered a more accurate description of the complex root morphology [7, 11]. A detailed research was carried out by Karobari et al. in 2020 in which they studied the root canal morphology of the anterior permanent dentition comparing two classification systems, and they reported that despite the wide range of variations in the canal morphology across different ethnicities and ages, the classification by Ahmed et al. reported accurate presentations [7, 12]. Another important research comparing the classification systems by Vertucci et al. and Ahmed et al. was conducted by Saber et al. in 2018, and they concluded that the latter had wider applicability and was more accurate than the former system [6, 7, 13].

## 3. Classification Systems

### 3.1. Weine's Classification

**Figure 1:**
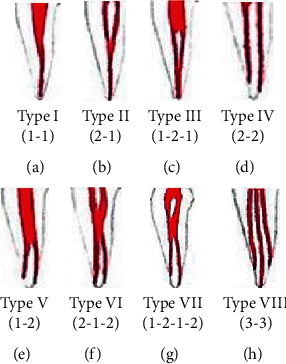
Weine's classification of root canal morphology from type I to type IV.

Root canal morphology was classified by Weine et al. [3, 4] from type I to type IV as follows (Figure 1):

Type I: a single main canal from the pulp chamber to the apex of the root

Type II: two separate canals starting from the pulp chamber and joining as one, just short of the root apex

Type III: two separate canals starting from the pulp chamber to the root apex

Type IV: a single canal starting from the pulp chamber and dividing into two canals near the root apex

### 3.2. Vertucci's Classification and Its Supplemental Configurations

**Figure 2:**
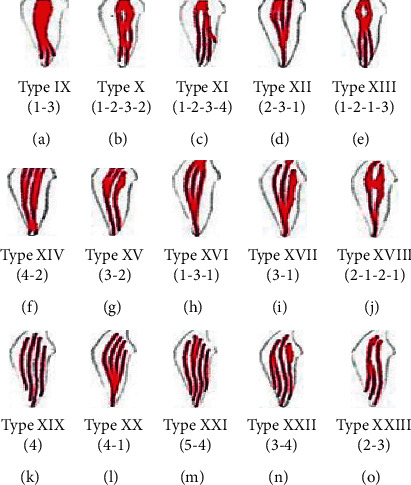
Vertucci's classification of root canal morphology from type I to type VIII.

Vertucci classified root canal morphology into eight types that are described as follows (Figure 2):

Type I: a single main canal is present starting from the pulp chamber to the root apex.

Type II: two separate canals leave the pulp chamber but join to form one canal to the apex.

Type III: one canal leaves the pulp chamber and divides into two smaller canals which later merge again to exit through one canal.

Type IV: two separate as well as completely distinct canals run from the pulp chamber to the root apex.

Type V: there is a single canal exiting the pulp chamber which divides into two canals with separate apical foramina.

Type VI: two separate canals join at the middle of the root to form one canal which extends till the apex, just short of the apex, and again divides into two.

Type VII: the canal starts as a single until the middle third of the root then divides into two separate canals that rejoin after some distance and then, near the apex, divides into two again.

Type VIII: the pulp chamber near the coronal portion divides into three separate canals extending till the apex [1, 5].

### 3.3. Sert and Bayirli [6] Added Supplementary Configurations to Vertucci's Classification System

**Figure 3:**
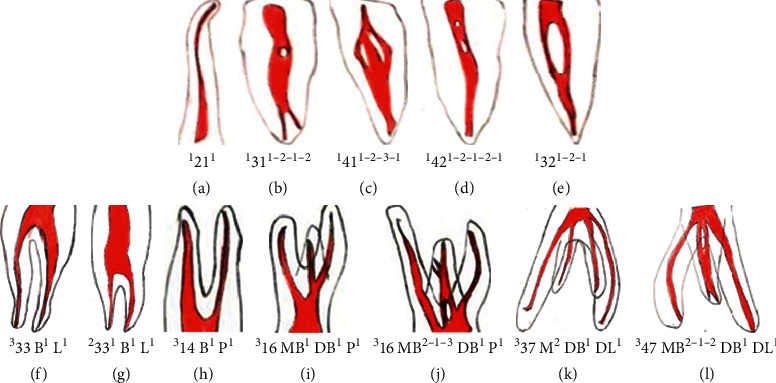
Supplemental configuration types to Vertucci's classification system for root canal morphology as suggested by Sert and Bayirli.

The authors evaluated the root canal configuration in 2800 maxillary and mandibular permanent teeth amongst Turkish population using a clearing technique. They added fourteen types to Vertucci's classification and numbered from Type IX to Type XXIII described as follows (Figure 3):

Type IX: a single canal starts from the pulp chamber and, during its course, divides into three.

Type X: a single canal starts from the pulp chamber and divides into two, out of which one canal further divides into two with two foramina.

Type XI: a single canal starts from the pulp chamber and divides into two, out of which one further subdivides into two and runs as three canals and ends with four foramina.

Type XII: two separate canals start from the pulp chamber, out of which one further subdivides into two and, later, all three join to form one canal with one foramen.

Type XIII: a single canal starts from the pulp chamber and divides into two canal which rejoins as one and further divides into three canals with three foramina.

Type XIV: four canals starts from the pulp chamber, and later, two of each will join and end with two foramina.

Type XV: three canals starts from the pulp chamber, out of which two join to form one canal and end with two foramina.

Type XVI: two canals start from the pulp chamber, out of which one further subdivides into two and ends with three foramina.

Type XVII: a single canal starts from the pulp chamber and divides into three canals which again rejoin to form a single canal with a single foramen.

Type XVIII: three canals start from the pulp chamber and rejoin to form a single canal with a single foramen.

Type XIX: two canals start from the pulp chamber and join as a single canal, further again divide into two, and rejoin as one canal with a single foramen.

Type XX: four canals start from the pulp chamber and end with four foramina.

Type XXI: four canals start from the pulp chamber and join as a single canal with a single foramen.

Type XXII: five canals start from the pulp chamber and one joins with another and ends as four canals with four foramina.

Type XXIII: three canals start from the pulp chamber, out of which one further divides into two and ends with four canals with four foramina.

Several studies have found root canal configurations to be highly complex and found nonclassifiable canal configurations during evaluation of internal and external anatomical canal variation using 3D imaging techniques [13–16]. Moreover, studies conducted by Filpo-Perez et al. and Karobari et al. found that 13% and 3% of samples, respectively, did not fit in Vertucci's classification and its supplements [17, 18]. In addition, the Vertucci classification does not consider the number of roots in the anterior and posterior teeth which is a major shortcoming.

### 3.4. The New Classification by Ahmed et al

**Figure 4:**
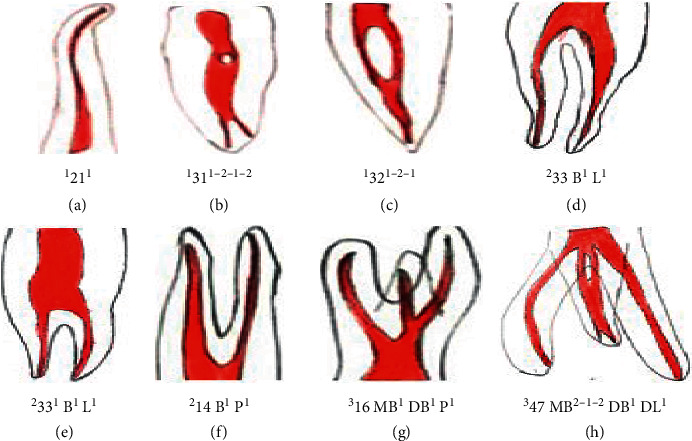
Classification of root and root canal morphology in permanent dentition by Ahmed et al.

Ahmed et al.'s classification system provides a single code for the tooth number, number of roots (considering any division of roots as two or more roots), and the canal configuration, hence giving a logical and accurate classification. One important facet of the new system is that teeth with the same canal configuration with separate roots are described as single code which reflects their anatomy accurately. It can also describe the previous nonclassifiable root canal morphologies with a simple single code giving an exact anatomy [19]. Furthermore, it overcomes the confusion of how to define the complex intercanal communications [20] (Figure 4).

## 4. Discussion

A new classification was introduced by Ahmed et al. [19]. This new system comprises of codes for tooth number, number of roots, and types of root canal morphologies. Recently, the authors explained the application and advantages of this new classification of root canal morphology in routine clinical practice and research [11, 21] and in primary dentition [10]. Commonly used classification systems given by Weine et al. and Vertucci et al. were useful in classifying many root canals, but not all canal morphologies [7, 10]. Furthermore, the need to remember classifications based on roman numericals is eliminated. Recently, a survey in Malaysia amongst final-year undergraduate dentistry students showed that more than 90% of students believed that the new system is more accurate and practical compared to the Vertucci classification [22].

The main aim of the classification was to create an easy, precise, and easily applicable classification of root canal morphology for the practitioners and researchers. The classification consists of three components, the tooth number, number of roots, and the number of root canals. Any tooth numbering system can be used to write tooth number such as the Federation Dentaire Internationale (FDI) system, universal numbering system, or any other. In extracted teeth, one can use abbreviations such as upper canine [UC] or lower lateral incisor [LLI]. The number of roots is added as a superscript before the tooth number [^R^TN]. The root canal configurations in each root (if more than one root) are added after the tooth number. Description of root canal configuration in each root will be given on the course of the root canal starting from the orifices [O], passing through the canal [C], and ending by the foramen [F], so it is like [TN^O−C-F^]. The classification for a single tooth will be written as [^R^TN^O−C-F^] [11]. Single-rooted, two-rooted, and three-rooted teeth can be classified using the new classification of root canal morphology, as shown in Figure 4 and Table 1. Ahmed et al.'s classification also has certain limitations. Multirooted posteriors with complex configuration, although can be presented accurately, will be presented with a long classification code. One of the important clinical implications of using the right classification system for canal configuration is that it could have a bearing on endodontic treatment as well as the fatigue resistance of rotary instruments. Cyclic fatigue resistance has been found to be linked and responsible for fracture of rotary endodontic instruments [23].

## 5. Conclusions

Sound knowledge of the root canal morphology is important for documentation and ease of communication amongst practitioners. All the classification systems noted till date have their own advantages and limitations. While Weine et al. classified only single-rooted teeth and did not classify multirooted teeth as well as complex configurations, Vertucci's classification further detailed complex configurations, with Sert and Bayirli adding supplemental types to the latter. Ahmed et al.'s classification system provides a single code for the tooth number and number of roots considering further division of roots as two or more roots, including the canal configuration; hence, it is considered a more logical and accurate classification.

## Figures and Tables

**Table 1:** Examples of different tooth morphologies following Ahmed et al.'s classification.

Figure 4	Classification code	Description
a	^1^12^1^	Maxillary right lateral incisor with a single root and a single root canal with one orifice and one apical foramen

b	^1^31^1−2-1-2^	Mandibular right central incisor with a single root having one canal initially with one orifice which later divides into two canals and rejoins as one and later divides into two with two apical foramina

c	^1^32^1−2−1^	Mandibular right lateral incisor with a single root initially starts as a single canal, and the orifice later divides into two canals and then rejoins as one canal with one foramen

d	^2^33B^1^L^1^	Mandibular left canine [33] with two roots, buccal and lingual, with two separate canals from the orifice with one foramen each

e	^2^33^1^B^1^L^1^	Mandibular left canine with two roots, buccal and lingual, with two canals having a single coronal canal

f	^2^14B^1^P ^1^	Maxillary right first premolar with two roots buccal and palatal root with a single root canal in each.

g	^3^16MB^1^DB ^1^ P^1^	Maxillary right first molar with three roots, mesiobuccal, distobuccal, and palatal, with each root having a single canal configuration

h	^3^47 M^2-1−2^ DB^1^ DL^1^	Mandibular right second molar with three roots; mesial root starts as two canals, joins to form one, and further divides into two canals and distobuccal root with single canal each

## Data Availability

Any required data shall be made readily available on request.

## References

[1] Vertucci F. J. (2005). Root canal morphology and its relationship to endodontic procedures. *Endodontic Topics*.

[2] Monsarrat P., Arcaute B., Peters O. A. (2016). Interrelationships in the variability of root canal anatomy among the permanent teeth: a full-mouth approach by cone-beam CT. *PLos One*.

[3] Weine F. S., Healey H. J., Gerstein H., Evanson L. (1969). Canal configuration in the mesiobuccal root of the maxillary first molar and its endodontic significance. *Oral Surgery, Oral Medicine, Oral Pathology*.

[4] Weine F. S., weine F. (1982). *Endodontic Therapy*.

[5] Vertucci F., Seelig A., Gillis R. (1974). Root canal morphology of the human maxillary second premolar. *Oral Surgery, Oral Medicine, Oral Pathology*.

[6] Sert S., Bayirli G. (2004). Evaluation of the root canal configurations of the mandibular and maxillary permanent teeth by gender in the Turkish population. *Journal of Endodontics*.

[7] Ahmed H. M. A., Versiani M. A., De-Deus G., Dummer P. M. H. (2017). A new system for classifying root and root canal morphology. *International Endodontic Journal*.

[8] Ahmed H. M. A., Che Ab Aziz Z. A., Azami N. H. (2020). Application of a new system for classifying root canal morphology in undergraduate teaching and clinical practice: a national survey in Malaysia. *International Endodontic Journal*.

[9] Ahmed H. M. A., Dummer P. M. H. (2018). A new system for classifying tooth, root and canal anomalies. *International Endodontic Journal*.

[10] Ahmed H. M. A., Musale P. K., El Shahawy O. I., Dummer P. M. H. (2020). Application of a new system for classifying tooth, root and canal morphology in the primary dentition. *International Endodontic Journal*.

[11] Buchanan G. D., Gamieldien M. Y., Tredoux S., Vally Z. I. (2020). Root and canal configurations of maxillary premolars in a South African subpopulation using cone beam computed tomography and two classification systems. *Journal of Oral Science*.

[12] Karobari M. I., Noorani T. Y., Halim M. S., Ahmed H. M. A. (2020). Root and canal morphology of the anterior permanent dentition in Malaysian population using two classification systems: a CBCT clinical study. *Australian Endodontic Journal*.

[13] Verma P., Love R. M. (2011). A Micro CT study of the mesiobuccal root canal morphology of the maxillary first molar tooth. *International Endodontic Journal*.

[14] Kim Y., Chang S.-W., Lee J.-K. (2013). A micro-computed tomography study of canal configuration of multiple-canalled mesiobuccal root of maxillary first molar. *Clinical Oral Investigations*.

[15] Lee K.-W., Kim Y., Perinpanayagam H. (2014). Comparison of alternative image reformatting techniques in micro-computed tomography and tooth clearing for detailed canal morphology. *Journal of Endodontics*.

[16] Leoni G. B., Versiani M. A., Pecora J. D., Damiao de Sousa-Neto M. (2014). Micro-Computed tomographic analysis of the root canal morphology of mandibular incisors. *Journal of Endodontics*.

[17] Filpo-Perez C., Bramante C. M., Villas-Boas M. H., Húngaro Duarte M. A., Versiani M. A., Ordinola-Zapata R. (2015). Micro-computed tomographic analysis of the root canal morphology of the distal root of mandibular first molar. *Journal of Endodontics*.

[18] Karobari M. I., Noorani T. Y., Halim M. S., Dummer P. M. H., Ahmed H. M. A. (2019). Should inter-canal communications be included in the classification of root canal systems?. *International Endodontic Journal*.

[19] Ahmed H., Adura Z., Azami N. (2020). Application of a new system for classifying root canal morphology in undergraduate teaching and clinical practice: a national survey in Malaysia. *International Endodontic Journal*.

[20] Karobari M. I., Noorani T. Y., Halim M. S., Dummer P. M. H., Ahmed H. M. A. (2019). Should inter‐canal communications be included in the classification of root canal systems?. *International Endodontic Journal*.

[21] Saber S. E. D. M., Ahmed M. H. M., Obeid M., Ahmed H. M. A. (2019). Root and canal morphology of maxillary premolar teeth in an Egyptian subpopulation using two classification systems: a cone beam computed tomography study. *International Endodontic Journal*.

[22] Ahmed H. M. A., Dummer P. M. H. (2018). Advantages and applications of a new system for classifying roots and canal systems in research and clinical practice. *European Endodontic Journal*.

[23] Miccoli G., Seracchiani M., Del Giudice A. (2020). Fatigue resistance of two Nickel-Titanium rotary instruments before and after ex vivo root canal treatment. *J Contemp Dental Pract*.

